# Which anatomical sites should be sampled for screening of MRSA carriage by culture or by rapid PCR test?

**DOI:** 10.1186/1753-6561-5-S6-O83

**Published:** 2011-06-29

**Authors:** DS Blanc, L Senn, I Nahimana, P Basset, G Zanetti

**Affiliations:** 1 Hospital preventive medicine, Centre Hospitalier Universitaire Vaudois, Lausanne, Switzerland

## Introduction / objectives

Rapid and accurate detection of MRSA carriage is a key element for therapy and for implementation of measures to prevent onward transmission. Nose is the anatomical site universally recommended for screening. However, other sites are also recommended, especially in countries with low MRSA incidence. Due to the high price of rapid PCR testing, it is important to know the value of testing each additional site.

## Objectives

To evaluate the value of each anatomical site for the screening of MRSA by culture and by a rapid PCR test.

## Methods

Screening samples included at least swabs of nose, throat, and groin. If applicable, others samples included swabs of wounds, catheterized urines, sputum, or others. Samples for culture were inoculated into an enrichment broth, which were plated after incubation onto chromogenic MRSA agar. Rapid PCR test were performed with GeneXpert MRSA.

## Results

12456 MRSA screening were performed by culture, among which 3137 (25.2%) had at least one MRSA-positive sample. The cumulative percentages of MRSA detection by culture increased from 48.1% for nose only, to 78.9 by adding groin, to 95.7 % by adding throat, and to 100% by adding other sites. These values were similar if the analysis was performed according to major MRSA genotypes, except a higher percentage of positive groin samples for the ST228-SCCmec-I clone.

2876 MRSA screening were performed by rapid PCR test, among which 312 (10.8%) had at least one positive sample. The cumulative percentages of MRSA detection increased from 61.9% for nose only, to 92.3 by adding groin, to 99.0 % by adding throat, and to 100% by adding other sites.

## Conclusion

Neither by culture nor by rapid PCR test is nose sampling sufficient for MRSA detection. Additional anatomical sites should include at least swabs from groin and throat.

## Disclosure of interest

None declared.

